# Evolution as a guide for experimental cell biology

**DOI:** 10.1371/journal.pgen.1007937

**Published:** 2019-02-14

**Authors:** Jeffrey Colgren, Scott A. Nichols

**Affiliations:** Department of Biological Sciences, University of Denver, Denver, Colorado, United States of America; New York University, UNITED STATES

The adherens junction is the main cell–cell adhesion structure in animal tissues. Core components of the adherens junction include cell-surface cadherin receptors and cytoplasmic effector proteins termed catenins (α-, β-, and p120-). These proteins interact to connect adjacent cells and tether points of cell–cell adhesion to the actin cytoskeleton. In addition to adhesion, the adherens junction has essential roles in cell-sorting during development, the establishment and maintenance of tissue polarity, mechanosensing and signal transduction, spindle orientation during cell division, and collective cell migration [1–6].

The adherens junction has been the subject of intensive research for more than three decades. Studies have progressed from ultrastructural descriptions using electron microscopy, to dissection of the molecular composition of the adherens junction, to characterization of its context-dependent functions and regulation. Following the first report on cadherin receptors in 1985, nearly 35,000 research articles and reviews have mentioned them by name. A literature search for β-catenin recovers more than 32,000 records since its discovery in 1989. What more is there to learn about so well-characterized a structure, and what methods will enable future discoveries?

**Fig 1:**
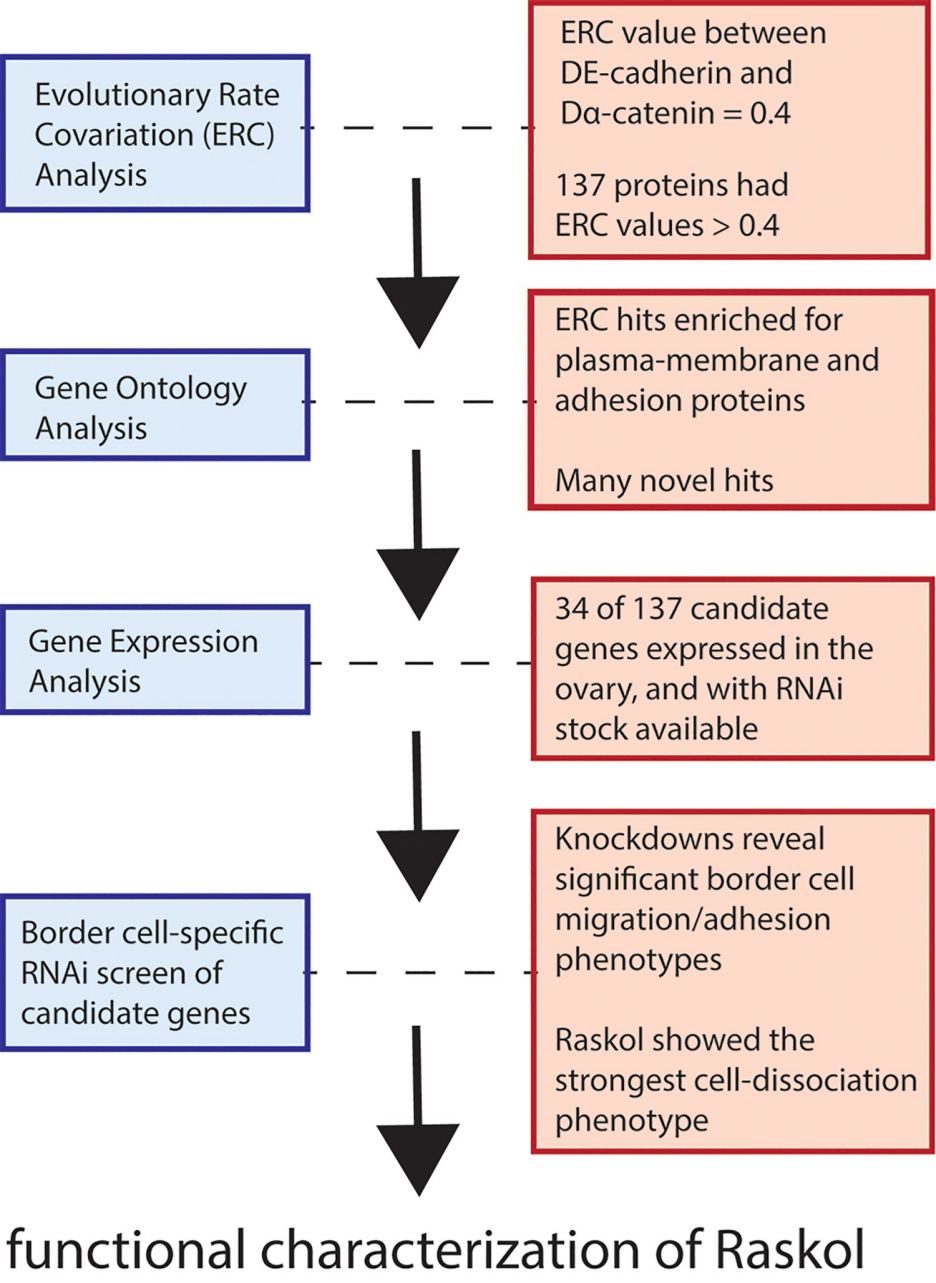
Workflow to screen for adhesion-related proteins involved in collective migration of *Drosophila* border cells. BC migration in the developing egg chamber of *Drosophila* is known to be regulated by DE-cadherin [8]. Raza and collegues used ERC analysis to identify proteins with evolutionary rates correlated with DE-cadherin. This analysis ultimately led to the discovery of Raskol—a putative GTPase activating protein that regulates DE-cadherin and actin dynamics in multiple tissues, including BCs. BC, border cell; ERC, evolutionary rate covariance; RNAi, RNA interference.

A study by Raza and colleagues [7] takes a fresh look at the adherens junction using a technique guided by evolutionary theory. Evolutionary rate covariation (ERC) analysis was coupled with traditional experimental techniques to reveal the GTPase activating protein (GAP) Raskol as a novel regulator of DE-cadherin (the *Drosophila* homolog of vertebrate E-cadherin) and actin dynamics during border cell (BC) migration in the *Drosophila* egg chamber. The ERC method was crucial for this discovery in that it narrowed the list of experimental candidates from the entire proteome to a short-list hypothesized to coevolve with DE-cadherin (Fig 1).

The ERC method relies on the principle that proteins with common functions will be subjected to similar selective pressures and will therefore exhibit correlated amino acid substitution rates [9–12]. On its surface, this method may seem overly simplistic. Out of the entire proteome, it is likely that some proteins will have similar evolutionary rates due to chance alone, whereas others will evolve at different rates despite having related functions. Indeed, the ERC method is not a catch-all. For example, Raza and colleagues found no evolutionary rate correlation between Armadillo (the *Drosophila* β-catenin ortholog) and DE-cadherin. This was expected because Armadillo also functions as a transcription factor in the Wnt signaling pathway, a role constrained by strong but different selective pressures. In contrast, positively correlated ERC values were detected between DE-cadherin with both α-catenin and p120-catenin, proteins with functions largely restricted to the adherens junction.

A strength of the ERC method is its ability to detect functional associations that may not show in traditional genetic screens or proteomic studies [12]. Raza and colleagues detected 137 proteins with ERC values correlated to DE-cadherin to the same degree as α-catenin. Of these, many were known regulators of cell adhesion or plasma membrane associated proteins but many were novel. There was little overlap between the positive ERC hits in this study with candidate adherens junction regulators detected in a recent genetic screen in Schneider 2 (S2) cells [13]. This discrepancy highlights the potential of the ERC method as a tool to aid in functional discovery and may be explained by the fact that functional interactions that are transient or highly context-specific are no less detectable by ERC than are abundant and stable interactions.

However, functional associations predicted by ERC fall far short of the standard of evidence for assessing gene and/or protein function. Positive hits must be experimentally validated. Raza and colleagues used an RNA interference (RNAi) screen to knockdown top ERC hits in the context of BC migration, a developmental process regulated by DE-cadherin [8]. By focusing on ERC hits that were expressed in the ovary, and for which RNAi stock were available, they narrowed their list to 34 candidates. Knockdown of proteins with higher ERC values resulted in stronger migration and adhesion phenotypes and frequently led to diminished DE-cadherin levels at BC boundaries.

The ERC hit with the strongest dissociation phenotype, which they named Raskol, had not been previously shown to be functionally linked to DE-Cadherin or adherens junctions in flies. By looking at colocalization of Raskol with DE-Cadherin, as well as further examining its knockdown phenotypes, they found strong evidence that it is a novel regulator of DE-Cadherin–dependent adhesion and is important during BC migration. In addition, they found evidence for its role as a regulator of actin dynamics. During BC migration, actin protrusions extend at the front of the cluster in the direction of migration. By knocking down Raskol, the authors observed these protrusions radiating indiscriminately from the migrating cluster, with no significant change in the occurrence of front-oriented protrusions.

The study by Raza and colleagues demonstrates an effective workflow to harness the predictive value of the ERC method to identify context-dependent functional interactions in cell and developmental processes. Given a resolved phylogenetic tree of closely related species, it is possible to create a comprehensive database of proteins with correlated ERC profiles. Powerful experimental techniques are available for the study of research organisms like *Drosophila*, but it is valuable to remember that cell and developmental processes are the product of evolution. Methods like ERC help to bridge the gap between evolutionary theory and experimental research. Recent work has considered lab animals in the context of their natural environments [14,15], and we gain much by considering lab animals in their phylogenetic context. In our opinion, the ERC method should be part of the toolkit of any experimental cell or developmental biologist.
